# Smartphone use disorder and future time perspective of college students: the mediating role of depression and moderating role of mindfulness

**DOI:** 10.1186/s13034-020-0309-9

**Published:** 2020-01-18

**Authors:** Yangchang Zhang, Shuai Lv, Cunya Li, Yang Xiong, Chenxi Zhou, Xuerui Li, Mengliang Ye

**Affiliations:** 1 Department of Epidemiology and Health Statistics, School of Public Health and Management, Chongqing Medical University, Chongqing, 400016 China; 2 The Innovation Centre for Social Risk Governance in Health, Chongqing Medical University, Chongqing, 400016 China; 3 School of Modern Logistics, Qingdao Harbour Vocational and Technical College, Qingdao, 266404 Shandong China; 4 Department of the First Clinical Medicine, Chongqing Medical University, Chongqing, 400016 China; 5 The First Affiliated Hospital of Chongqing Medical University, Chongqing, China

**Keywords:** Future time perspective, Smartphone use disorder, Mindfulness, Depression

## Abstract

**Background:**

Smartphone use disorder (SUD) of college students has drawn increasing attention. Although future time perspective (FTP) may be an important protective factor for individual SUD, the moderating and mediating mechanisms underlying this relationship remain unknown. We tested the individual roles of depression and mindfulness as moderators of this relationship.

**Methods:**

A cross-sectional study was conducted in two colleges in Shandong and Chongqing in China using a sample of 1304 college students recruited by stratified cluster sampling. Data were collected through a validated self-report instrument. A moderation–mediation model was constructed, and an SPSS PROCESS macro was used to analyse the data.

**Results:**

The correlation analyses showed that FTP was negatively associated with SUD of college students. The mediation model revealed that depression partially mediated the link between FTP and SUD of college students. The moderation–mediation model suggested that mindfulness moderates two direct paths: FTP to depression and depression to SUD. In the first path (FTP to depression), a high level of mindfulness among college students had weakened the relationship between FTP and depression. Here, the relationship is strengthened by a low level of mindfulness. In the second path (depression to SUD), low levels of mindfulness strengthen the link between depression and FTP. In contrast, significant association was not found with high levels of mindfulness.

**Conclusions:**

Results suggest that interventions, such as improving the individual level of FTP and mindfulness, should be conducted. These interventions, in turn, help control the level of depression in college students and ultimately decrease their level of SUD.

## Background

Mobile phones, especially smartphones, are widely used and popular with adolescents worldwide. Compared with traditional mobile phones, smartphones are superior with their numerous functions that include communication and entertainment. According to the China Internet Information Centre survey in 2018, the number of smartphone users has rapidly increased to 98.6% among the Internet-using population, as registered users of smartphones had exceeded more than 829 million in China [1]. Notably, ‘gaming disorder’ (GD) has recently been defined as a mental disorder due to excessive gaming behaviour and even deemed to be an addiction. This notion was approved by the World Health Organization in the 11th Revision of the International Classification of Disease (ICD-11). On the basis of the ICD-11, smartphone use disorder (SUD) may be a potential kind of GD and may have attracted major concerns in society [2, 3]. SUD is often accompanied by a series of physical and mental health problems, such as GD, sleep disorders, poor concentration, negative emotions including anxiety and depression [4, 5]. Young people and college students with SUD often exhibit antisocial behaviours, such as poor peer relationships and suicidal ideation, and are often involved in crimes [6, 7]. These pathological behaviours increase the odds of early death among adolescents. Moreover, SUD and Internet communication disorder (ICD) are related with substance addiction. Chamberlain et al. found that SUD was associated with alcohol use disorder via large-scale email investigation [8]. Moreover, Montag et al. reported that the association between ICD and alcohol consumption was moderated by smoking status, and people who quit smoking may have a tendency to increase Internet overuse and consumption of mixed alcoholic beverages [9]. To further understand and develop these new psychological mechanisms about SUD and related risk factors, temperament or personal psychological traits were further observed and studied [10–12].

Cognitive–behavioural theory of Davis is known as a broad interpretation and understanding of the etiology, development and outcomes of ICD based on a model of pathological Internet use [13]. Davis suggested that maladaptive cognition is the potential instigator of Internet addiction (IA), which induces relative emotional and behavioural symptoms. Recently, the Interaction of Person-Affect-Cognition-Execution (I-PACE) model was regarded as an important theoretical framework, which integrates individual psychological and neurobiological aspects of ICD. Many significant characteristics, such as psychopathology, vulnerable personality traits (P), affective (A) and cognitive (C) responses to Internet use experience of users, executive and inhibitory control and decision-making behaviour, may all lead to the addictive use of a specific Internet application [3]. In 2019, an updated version of the I-PACE model was found to be effective for several types of addiction behaviours including gambling, buying–shopping, compulsive sexual behaviour disorders and gaming [14]. Empirical studies have indicated that future time perspective (FTP) and mindfulness are important cognitive factors affecting ICD [15, 16]. Although SUD has many similarities with behaviour addiction (e.g. ICD and pathological gambling) in its symptoms and adverse consequences, scholars suggest that SUD belongs to substance addiction [17]. Hence, FTP and mindfulness may also be key elements in the development of SUD. Time perspective (TP) is a personality trait of individual cognition, experience and action (or action tendency) about time [18]. FTP is separated from TP, and this separation implies that individuals have relatively stable personality characteristics about the future. Thus, FTP is considered a potential protective factor against ICD [19, 20]. Expectancy–value theory of Wigfield reasons that outcome expectancy and value assessment decide individual behavioural motivation [21]. FTP can influence individual expectancy or value regarding the choice to delay gratification [19]. That is, FTP promotes individuals to pursue high-level value in the future and devalue immediate gratification. In addition, Park et al. reported that FTP of female high-school students was negatively associated with smartphone addiction [22]. This negative relationship was found again among college students in mainland China [23]. Thus, people with high-level FTP may have a lower risk of SUD than those with low-level FTP.

Karel and Erin argued that personality traits are closely related to mental health, such as depression in college students, and are essential elements for individual well-being and health behaviour [24]. As an important personality trait, TP could be associated with many psychosocial variables (e.g. depression, self-esteem) [25]. The broad Big Five of Personality is regarded as a consistent and stable model in describing different personality traits. Overall, the broad Big Five of Personality have direct and indirect effects on addictive behaviours, such as smoking, drinking and Internet use [26]. Specifically, agreeableness can predict personal SUD/ICD and present negative direct effect because a low level of agreeableness is associated with high SUD/ICD [27]. Agreeableness mainly includes trust, honesty, avoidance and compliance. Individuals with low agreeableness tend to be self-centred and competitive. They are uncooperative and unwilling to comply to rules and regulations [28]. Hence, individuals with low level of agreeableness are prone to addictive behaviours. Similarly, people with short-sightedness do not always believe in the instrumental relationship between their behaviour and future time, leading to an unrealistic optimism about the future. From this point of view, TP may be linked to agreeableness, and both contribute to the outcome of SUD/ICD. In line with this notion, well-executed research with diverse methodologies and population have confirmed that TP could predict individual depression. For example, a mixed methods study using qualitative and quantitative methods was conducted to examine levels of individuals with alcohol/drug dependence, and a positive association was found among past negative TP and present hedonistic TP and depression [29]. In addition, an observational study using the Berlin Aging Study revealed a significant relationship between high depressive symptoms and low FTP, which could be explained by poor health behaviour and low adaptive coping [30, 31]. Furthermore, an empirical study controlling the influence from psychological factors of parents indicates a potential causation between great FTP of adolescents and low level of depressive symptoms [32]. Results indicate that the risk dimension of TP can play a negative role and that FTP as a protective factor, conversely, may mitigate adverse mental factors. Psychosocial factors have been keys to hypothesise the maintenance and development of SUD. Considerable research regarding the association and result between depression and SUD have been widely studied and reported from different areas and cultural backgrounds [11, 33–35]. Although an enormous amount of research is in line with the notion that high depression could predict adolescent SUD, studies that have directly tested the mediating role of depression in the association between FTP and SUD are minimal. For example, Montag et al. found that maladaptive cognitive and emotion process were significant mediators between anxiety and depression with SUD [36]. Individuals with high depression feel that they are useless and lose control of their lives. However, Internet gaming has characteristics, such as strong operability, positivity and instant feedback. Players can develop feelings of strength and achievement in a short time. Therefore, individuals with high depression consider that Internet gaming alleviates the threat of self-value; hence, they can experience their identity in the virtual environment instead of the real world, resulting in maladaptive cognition.

Individual traits are influenced by age, sex, emotion, experience, interpersonal relationships and the environment [37]. Given this complexity, FTP research presents inconsistent results between different addiction behaviours. For example, high-level FTP can decrease cigarette and drug use among high school students, but this association was not found in people with drug and alcohol addictions [38]. This inconsistency indicates that the generative mechanisms of behaviour and substance addiction may be distinct. One explanation is that individual FTP varies from person to person, influencing their feedback for risk perception, and FTP is considered a dynamic process [39, 40]. Results indicate that cognitive–behavioural theory had successfully explained part of addiction behaviour, but more addict behavioural variances have yet to be explained fully. The reason for this lack of explanation is that other potential variables exist between behavioural intention and behavioural occurrence that affect individual specific behaviour. Hence, the above evidence supports the moderating effect of FTP and SUD.

Mindfulness is defined as a meta-cognitive state in which individuals focus on present-moment experiences without judgment or evaluation [41, 42]. Mindfulness is also considered a personality trait of perceived emotion and behaviour [43]. High-level mindfulness means that individuals adopt an open and receptive attitude to current experiences, which effectively offsets the effect of stressors [42]. When dealing with stressors, excessive attention to the past or future (e.g. TP) can lead to depression and anxiety [42]. In addition, mindfulness refers to the tendency to remain alert in daily life, which is positively associated with better sleep quality, well-being, life satisfaction, negatively associated anxiety, depression and impulsiveness [44–47]. Empirical studies show that non-clinical mindfulness training can improve positive emotional level and buffer negative emotions of individuals [48, 49]. Hence, mindfulness may play a moderator role in the relationship between TP and emotion.

Current or past beliefs may impact individual future awareness and behaviour. Mindfulness is used to evaluate the focus of individuals on experiences at a particular moment, and these experiences include a focus on *now* without consideration of future external events, emotions, thoughts or intentions [50]. In a cross-sectional study from the USA, mindfulness was found to significantly predict individual promotion focus and prevention focus, and this link was strongly associated with prevention focus [51]. According to Higgins, individuals with promotion focus tend to pursue ideal goals and increase self-perception, which decrease the control of external safety and protection. This notion negatively impacts attention to the future and positively increases the odds of risk behaviour [52]. In contrast, individuals with prevention focus tend to purse the goal of responsibility and increase rational cognition. They believe that inaction will maintain the status quo, and while reducing the expectation of benefits, they become increasingly concerned about the benefits after use [52]. This notion positively impacts attention to the future and is negatively linked with the odds of risk behaviour. Hence, mindfulness may be a potential moderator influencing risk behaviour and attention to future with change in *now* focus. In a repeated-measures web survey, researchers found that distress tolerance and mindfulness were inversely associated with levels of SUD, and mindfulness can partly explain the mechanism between depression and anxiety sensitivity with the level of SUD [53].

Hundt found that promotion focus could positively influence drug addiction and drinking behaviour, and prevention focus could negatively influence drug addiction [54]. Different conditional levels exist among individuals with the same addiction behaviour. Individuals with high levels of mindfulness may have excellent conditional ability, such as promotion and prevention focus, and low levels of mindfulness would weaken this efficiency. In our study, people with low focus are always more frequent mobile phone users, which leads to SUD [55]. The hypothesis of mindfulness as a moderator between FTP and SUD was further enhanced.

To date, only a few researchers have examined mindfulness as a moderator of the indirect relationship between FTP and SUD of college students. We expect our study to provide in-depth understanding of IA among college students and to contribute to the development of effective mental health interventions. The hypotheses of this study are as follows:

### Hypothesis 1

FTP is negatively correlated with the depression variable but is positively correlated with SUD. That is, the depression variable mediates the link between FTP and SUD of college students.

### Hypothesis 2a

Mindfulness moderates the association between FTP and depression or depression and SUD.

### Hypothesis 2b

The indirection between FTP and SUD through depression varies as a function of mindfulness.

### Hypothesis 3a

The indirect relationship between depression and SUD of college students is strong with low levels of mindfulness and is weak with high levels of mindfulness.

### Hypothesis 3b

The indirect relationship between FTP and depression is weak with high mindfulness and strong with low mindfulness.

**Fig. 1:**
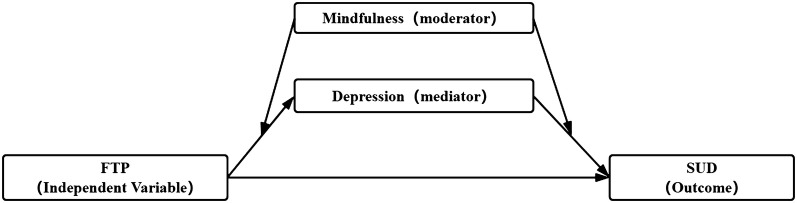
Conceptual diagram of the moderation–mediation model. *FTP* future time perspective, *SUD* smartphone use disorder

Considering the purposes of this study, a moderation–mediation model was constructed and is depicted in Fig. 1.

## Methods

### Participants

A cross-sectional study was conducted from March to April 2019 in Qingdao, Shandong and Chongqing in China. A total of 1304 freshmen and sophomore student participants were recruited from two colleges in Qingdao and Chongqing by stratified cluster sampling. The mean age of the participants was 19.71 years (SD = 1.03, range = 18 to 22 years), and 522 (40%) of respondents were male. Before the experiment, we provided a detailed explanation about the purpose and methods of the study and requested oral and written informed consent from participants. Self-reported questionnaires were then distributed in class to collect data on age, sex and related research variables from students. At the end of the investigation, we offered small gifts to participants.

### Measures

#### Future time perspective

The 28-item FTP scale of Young was used to test the levels of FTP of participants [56]. This scale mainly measures cognition, experience and behavioural tendency of adolescents towards future time (e.g. ‘As long as it is conducive to my own development, I can persist in overcoming difficulties and accomplishing tasks’). The FTP scale of Young, as a Chinese version, has been commonly used among Chinese adolescent students [57, 58]. Participants rated each item on a five-point scale that ranged from one (never) to five (always). Two dimensions, future negative and future confused, were reversely scored. High total scores indicated high levels of FTP. This scale was highly reliable (*Cronbach’s α *=* 0.90*).

#### Smartphone use disorder

The mobile phone addiction index (MPAI) was applied to test adolescent SUD [59]. This scale consists of 17 items (e.g. ‘If you have not checked messages or turned on your cell phone for a while, you will feel anxious’). Participants rated each item on a five-point scale that ranged from one (never) to five (always). The Chinese version of MPAI reveals adequate psychometric properties among Chinese adolescents [60, 61]. All items are forward scored. High total scores indicate high possibility of SUD. This scale demonstrated high reliability (*Cronbach’s α *=* 0.90*).

#### Depression

The Depression Anxiety Stress Scale-21 was used to check the mental condition of participants, including depression, anxiety and stress [62]. In the present study, the Chinese version of this scale was used to test depression of students [63]. This scale consists of seven items to evaluate the level of depression (e.g. ‘I find working up the initiative to do things difficult’). Participants rated each item on a five-point scale that ranged from one (always inconsistent) to five (always consistent). High scores indicate a high level of depression. This scale reported high reliability (*Cronbach’s α *=* 0.91*).

#### Mindfulness

The Child and Adolescent Mindfulness Measure (CAMM) is a six-point scale used to assess mindfulness of an individual [64]. The scale is confirmed to have good psychometric properties [65]. This scale consists of 10 items (e.g. ‘I get upset with myself for having feelings that do not make sense’). Participants rated each item on a five-point scale that ranged from zero (never) to four (always). The Chinese version of the CAMM shows satisfactory test–test reliability and validity among Chinese adolescents [66, 67]. All items were conducted with reverse scoring. High scores indicate a high level of mindfulness. This scale showed high reliability (*Cronbach’s α *=* 0.82*).

### Statistical analyses

Statistical analyses were conducted using SPSS 23 (SPSS Inc., Chicago, IL, USA). Firstly, a correlation matrix of Pearson that included FTP, SUD, depression and mindfulness was developed. Secondly, to identify the mediating effect of depression, we used the four-step procedure of MacKinnon to test (1) the significance of the relationship between FTP and SUD, (2) the significance of the association between FTP and depression, (3) the significance of the association between depression and SUD while controlling for FTP and (4) the significance of the coefficient for the indirect path between FTP and SUD through depression (Hypothesis 1) [68]. Thirdly, we examined whether mindfulness moderates the direct effect of FTP and depression or depression and SUD (Hypothesis 2a). Fourthly, we identified the overall moderated mediation (Hypothesis 2b). Finally, simple slope tests (mean ± SD) were conducted to demonstrate the relationship between FTP and depression and depression and SUD among adolescents at low and high levels of mindfulness (Hypotheses 3a and 3b). All continuous variables were mean-centred before constructing the moderation–mediation model to avoid multicollinearity. Hayes’ SPSS PROCESS macro (Model 58) was applied to construct this moderation–mediation model with age and sex controlled [69]. The bootstrapping technique was used to estimate 5000 resamples of the data, and 95% bias-corrected confidence intervals (CIs) were calculated and checked. Supposing that confidence intervals did not include zero, the effects indicated significant levels.

## Results

### Correlations between the main study variables

Pearson correlation coefficients for the main study variables were calculated and are presented in Table 1. FTP was positively correlated with mindfulness (*r* = 0.35, *p* < 0.01) and negatively correlated with depression (*r* = − 0.60, *p* < 0.01) and SUD (*r *= − 0.40, *p* < 0.01). Mindfulness was negatively correlated with depression (*r* = − 0.57, *p* < 0.01) and SUD (*r* = − 0.53, *p* < 0.01). Participants with high levels of depression were inclined to develop SUD (*r* = 0.46, *p* < 0.01). Table 2 shows the descriptive analysis of study variables by gender.

**Table 1:** Correlations between the main study variables

Variables	M	SD	FTP	Mindfulness	Depression	SUD
FTP	100.26	15.30	1			
Mindfulness	37.54	5.83	0.35**	1		
Depression	12.66	4.76	− 0.60**	− 0.57**	1	
SUD	33.89	9.88	− 0.40**	− 0.53**	0.46**	1

*M* mean, *SD* standard deviation, *FTP* future time perspective, *SUD* smartphone use disorder

* *p *< 0.05, ** *p *< 0.01, N = 1304

**Table 2:** Descriptive analysis of study variables by gender

Variable	Gender		*p* value
	Male (mean ± SD)	Female (mean ± SD)	
Age	19.96 ± 1.18	19.65 ± 1.04	< 0.001
FTP	100.51 ± 16.69	100.19 ± 14.83	0.75
Mindfulness	36.89 ± 6.47	37.76 ± 5.60	< 0.05
Depression	12.96 ± 5.02	12.56 ± 4.67	0.21
SUD	32.02 ± 10.91	34.49 ± 9.46	< 0.001

### Testing of the mediation model

In Hypothesis 1, the depression variable mediates the link between FTP and SUD of college students. To verify the assumption, multiple linear regression was conducted to test the effects of mediation. In the first step, FTP was negatively associated with SUD in Model 1 (*β* = − 0.26, *p* < 0.001). In the second step, FTP was negatively associated with depression in Model 2 (*β* = − 0.19, *p* < 0.001). In the third step, depression was positively associated with SUD when the direct effect of FTP on SUD was controlled in Model 3 (*β* = 0.696, *p* < 0.001). In the fourth step, the bootstrapping technique and 95% bias-corrected confidence intervals were calculated to test the mediating effect. Results showed that depression partially and significantly mediates the relationship between FTP and SUD (*ab* = − 0.13, *SE* = 0.02, 95% CI = [− 0.16, − 0.10]). The mediation effect accounted for approximately 50% of the overall mediation model. As these four steps were all significantly established, Hypothesis 1 is supported (Tables 3 and 4).

**Table 3:** Regression results for mediating test

Predictors	Model 1: SUD			Model 2: Depression			Model 3: SUD		
	*β*	*SE*	*t*	*β*	*SE*	*t*	*β*	*SE*	*t*
FTP	− 0.26	0.02	− 15.60***	− 0.19	0.01	− 26.93***	− 0.13	0.02	− 6.48***
Depression							0.70	0.06	11.08***
*R*^*2*^	0.49			0.36			0.24		
*F*	200.63***			752.02***			200.63***		

*FTP* future time perspective, *SUD* smartphone use disorder

*** *p* < 0.001, *N* = 1304

**Table 4:** Total, direct and indirect effects of FTP on SUD

Item	*β*	*SE*	*95% CI*
Total effect of FTP on SUD	− 0.26	0.02	(− 0.29, − 0.23)
Direct effect of FTP on SUD	− 0.13	0.02	(− 0.09, − 0.01)
Indirect effect of FTP on SUD	− 0.13	0.02	(− 0.16, − 0.10)

*N* = 1304

### Testing of moderation

In Hypotheses 2a and 2b, we assumed that mindfulness moderates the indirect association between TP and SUD. The PROCESS macro (Model 58) was used to test the moderation–mediation model. In this study, two regression models were constructed, and regression coefficients were estimated. In Model 1, we examined the moderating effect of mindfulness on the relationship between TP and SUD. In Model 2, we tested the moderating effect of mindfulness on the relation between depression and SUD. Table 5 presents the estimated moderating effects of the two models.

**Table 5:** Testing the moderated mediation effect of FTP and SUD

Predictors	Model 1: Depression			Model 2: SUD		
	*β*	*SE*	*t*	*β*	*SE*	*t*
FTP	− 0.15	0.01	− 22.94***	− 1.13	0.02	− 7.21***
Depression				0.19	0.07	2.79**
Mindfulness	− 0.32	0.02	− 18.89***	− 0.66	0.05	− 14.25***
FTP × mindfulness	0.01	0.01	8.03***			
Depression × mindfulness				− 0.02	0.01	− 2.43*
*R*^*2*^	0.53			0.34		
*F*	488.85***			169.15***		

* *p* < 0.05, ** *p* < 0.01, *** *p* < 0.001, *N* = 1304

Table 4 shows that the total effect of FTP on SUD was significant (*β* = − 0.26, 95% CI = − 0.29, − 0.23), and the indirect effect of mediation was supported (*β* = − 0.13, 95% CI = − 0.16, − 0.10). According to the results of Model 1 in Table 5, the effect of FTP on depression was significant (*β* = − 0.15, *p* < 0.001), and this effect was moderated by mindfulness (*β* = 0.01, *p* < 0.001). In Model 2, the main effect of depression on SUD was significant (*β* = 0.29, *p* < 0.001), and this association was moderated by mindfulness (*β* = − 0.02, *p* < 0.05). Hence, Hypotheses 2a and 2b are supported.

**Fig. 2:**
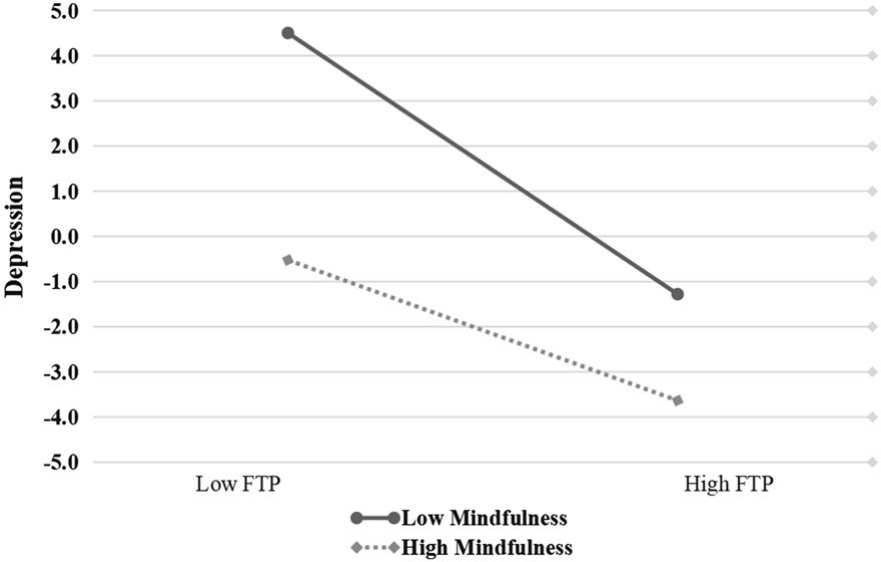
Depression among college students as a function of FTP and mindfulness (*N* = 1304)

**Fig. 3:**
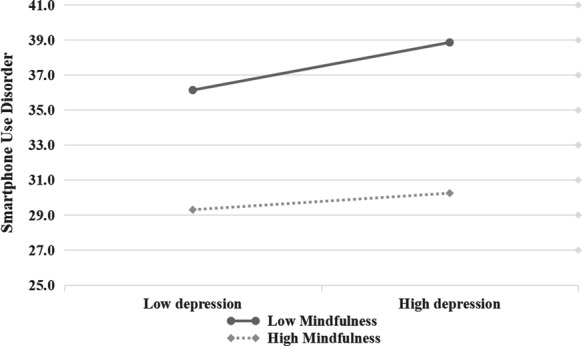
SUD among college students as a function of mindfulness and depression (*N* = 1304)

To clearly explain the essence of the interaction effect in the moderation–mediation model, the levels of mindfulness were divided into high and low groups (mean ± SD), and simple slope tests were used to show the effect trend of FTP on depression and depression on SUD under different mindfulness levels (Hypotheses 3a and 3b). As Fig. 3 depicts, participants with high levels of mindfulness and FTP were associated with low levels of SUD (*β*_sample_ = − 0.10, *p* < 0.001). This significant association was also supported by low levels of mindfulness (*β*_sample_ = − 0.19, *p* < 0.001), as shown in Fig.2. Adolescents with low levels of mindfulness and high levels of depression were positively associated with SUD (*β*_sample_ = 0.29, *p* < 0.001). However, this trend was not supported by high levels of mindfulness (*β*_sample_ = 0.10, *p* > 0.05), as shown in Fig. 3. Thus, Hypothesis 3b is fully supported, and Hypothesis 3a is partially supported.

## Discussion

Previous studies provided evidence of the negative association between FTP and SUD. However, the role of FTP and SUD as mediating and moderating mechanisms remain unclear. In the present study, FTP negatively predicts depression and depression positively predicts SUD. This indirect relationship is moderated by mindfulness in a two-stage mediation process. Specifically, high levels of FTP as a protective factor of depression are moderated by increased mindfulness, which was observed in adolescents with low levels of mindfulness. On the other paths, high levels of depression, as a risk factor of SUD, are moderated by increased mindfulness, but this effect was found in adolescents with low levels of mindfulness. Overall, our study provides a promising research approach for examining the association between FTP and SUD.

A direct effect was found between FTP and SUD. Firstly, FTP had a negative predictive effect on SUD, and these results are consistent with those reported in previous studies [12, 39]. Low levels of FTP indicate a significant association with fatalism and pessimism [70]. People with low levels of FTP only focus on the past and present and lack a proper plan for their own behaviour; they are inclined to instant gratification and impulsive behaviour [70]. In contrast, individuals with high levels of FTP have better self-regulation and make long-term plans with purpose. With full consideration of consequences, they make rational decisions to achieve long-term benefits [71]. Neurobiological research has reported that TP mainly activates individual anterior cingulate cortex, temporal cortex, parietal lobe and prefrontal cortex. In particular, FTP activates the ventral prefrontal cortex [72]. When individual impulsion and reflection are unbalanced, the prefrontal cortex reacts abnormally, resulting in behavioural addiction. Problematic use of social media/messenger app was connected with the change of neurobiological function. Individuals with high levels of addictive social app use were accompanied with low volume of grey matter in the subgenual anterior cingulate and the nucleus accumbens [73, 74]. Scholars also found that SUD is associated with cortical areas in the brain due to the use of touch display for a long time with fingers [75]. Hence, individuals with low levels of FTP may increase their risk of SUD due to the hyperactivation of cortical areas in the brain, which influence the reward and cold-executive functions.

Secondly, a mediating effect of depression was found between FTP and SUD. On the basis of theory of ‘diathesis–stress’ in depression, low stress can trigger depression in individuals with high susceptibility [76]. This theory reflects individual differences in internal diatheses, such as self-esteem and personality [77]. As a stable personality trait, FTP can also have a different influence on individual emotions. In this study, college students with high levels of FTP were likely to have good mental status. This result corresponds with that of a previous study wherein past negative and current perspectives were explored to test the relationship between depression and anxiety [33]. Regarding the association between depression and SUD, depression is positively associated with SUD. This result is congruent with the findings reported in previous theoretical and empirical research [33, 78] and is supported by theory of social support [79]. The theory indicates that people with depression would seek social support to fight depression and that smartphones can provide instant interpersonal interaction to alleviate negative emotions. By using mobile phones, participants experience social support or pleasure, which can be considered types of reward. On the basis of operational conditional reflex [80], people who lack social support increase the duration and frequency of mobile phone use to gain rewards, which, in turn, aggravate mobile phone addiction.

Thirdly, the moderating effect of mindfulness is significant between FTP and SUD. Results suggest that mindfulness can predict SUD of college students. College students with low levels of mindfulness are prone to SUD. Our findings are consistent with those of a previous study that indicated that individuals with a high level of SUD possess weak inhibition [81]. Mindfulness can help develop individual positive coping techniques for needs, namely, observation without reaction, which can prevent the automatic connection between needs and behaviour. Finally, in individuals who show decreased needs for stimulus, addiction behaviour decreases, and inhibition ability is promoted.

Mindfulness can moderate the path between FTP and depression. The effect of FTP on depression was negative in college students with a high level of mindfulness and was heightened with low levels of mindfulness. Mindfulness refers to focusing on the present moment in a conscious and non-critical way, which can promote the individual support of psychological resources. For example, high levels of mindfulness in college students may enhance levels of hope and strengthen psychological resilience and self-efficacy [82, 83]. As FTP indicates positive psychological resources for high levels of mindfulness in college students, the interaction effect of FTP and mindfulness may increase the individual ability to buffer mental problems. The hypothesis based on general strain theory indicates that when individuals feel stress and strain, they experience negative emotions that induce inadaptable behaviour [84]. Students with low levels of mindfulness have a poor ability to control and regulate impulsive emotions and behaviour and have a poor awareness of FTP [85]. This scenario could increase the risk of negative emotions caused by poor FTP. In contrast, people with high levels of mindfulness have a low probability of suffering from poor FTP, and the effect of moderation was weakened in this path (FTP to depression). In the mechanism of protective and risk factors [86], low levels of mindfulness play a risky role among college students. Mindfulness strengthens the prediction of depression by poor FTP and mainly plays a compensatory role in college students with high levels of mindfulness to offset and compensate the influence of FTP on depression.

Moreover, the relationship between depression and SUD was significant in college students with a low level of mindfulness but was not significant for college students with high levels of mindfulness. According to the re-perceiving model, ‘re-perception’, as a key element of mindfulness, can help individuals objectively experience internal and external stimuli and reduce the subjective feeling of adverse stimuli, and in turn tolerantly accept internal and external experiences [87]. Individuals with a high level of mindfulness are able to concentrate on the present and worry about the past and future without negative emotions. Thus, the mediating relationship between depression and SUD is not significant for them. Individuals with low levels of mindfulness possess poor inhibition functioning and self-control and they are always concerned with the past and future, resulting in depression and anxiety [82]. People with low levels of mindfulness may have high levels of cortisol, which influences individual stress reactions [88]. In addition, people with low levels of mindfulness become impulsive, and the buffer effect of mindfulness for anxiety and depression decreases [89]. Therefore, mindfulness can be regarded as an indicator when testing individuals for negative emotions, such as depression, and can prevent mobile phone addiction.

In the current study, identification of SUD requires more objective measurement index than self-reported data. The majority of mobile applications are designed to prolong usage time leading to individual time distortion. Some scientists and technicians attempted to develop new techniques to measure and analyse smartphone addiction [90–92]. For example, Lin et al. identified smartphone addiction via app-generated parameters, and the results suggested that recorded total usage time via app was significantly higher than self-reported usage time because of the time distortion effect [90]. However, Montag et al. found that total length of weekly mobile phone usage in hours was overestimated and weekly outgoing calls were underestimated [91]. Furthermore, researchers reported that social media/messenger and freemium game applications were significant drivers of excessive smartphone usage; these applications could be much more addictive than traditional/classic computer/console games [92]. Hence, enterprises should seriously consider how to develop less addictive products and recognise what kinds of approaches facilitate use reduction and create the healthiest effects.

The present study has several limitations. Firstly, as a cross-sectional study, it cannot reveal the causal relationship between variables. Follow-up studies or experimental methods are needed to verify the relationship between variables. Secondly, study variables were collected by self-reporting, in which retrospective memory bias is possible. Future research can adopt objective and effective indicators. Thirdly, personality traits, such as psychological resilience, may also affect SUD of college students. Further research can test the association between different personality traits and SUD. Fourthly, we conducted this study only in two colleges in Chongqing and Shandong, and the conclusion lacks extrapolation. Further research should be developed on a large sample and other age groups. Finally, this study included more female than male students, and maladaptive cognitions may indicate differences between sexes.

## Conclusions

FTP can be a protective factor for SUD. The mediation analysis indicated that depression can be a potential mechanism underlying this relationship. Moderated mediation indicated that mindfulness moderates the relationship between FTP and SUD, and college students with low levels of mindfulness are likely to be affected by depression and engage in addictive behaviour. This likelihood demonstrates the superposition effect of the two-risk factor. Results suggest that interventions increase the level of FTP and mindfulness, which in turn decrease the level of depression of college students and ultimately their SUD.

## Data Availability

The datasets used and analysed in the current study are available from the corresponding author on reasonable request.

## Abbreviations

FTP: future time perspective
SUD: smartphone use disorder
ICD: internet communication disorder
GD: gaming disorder

## References

[1] China Internet Information Center. The 43rd statistical report on the development of internet in China. http://www.cnnic.net.cn/hlwfzyj/hlwxzbg/hlwtjbg/201902/t20190228_70645.htm. Accessed 1 May 2019.

[2] Panova T, Carbonell X (2018). Is smartphone addiction really an addiction?. J Behav Addict.

[3] Brand M, Young KS, Laier C, Wölfling K, Potenza MN (2016). Integrating psychological and neurobiological considerations regarding the development and maintenance of specific Internet-use disorders: an Interaction of Person-Affect-Cognition-Execution (I-PACE) model. Neurosci Biobehav Rev.

[4] Kim SE, Kim JW, Jee YS (2015). Relationship between smartphone addiction and physical activity in Chinese international students in Korea. J Behav Addict.

[5] Lemola S, Perkinson-Gloor N, Brand S, Dewald-Kaufmann JF, Grob A (2015). Adolescents’ electronic media use at night, sleep disturbance, and depressive symptoms in the smartphone age. J Youth Adolesc..

[6] Wang P, Zhao M, Wang X, Xie X, Wang Y, Lei L (2017). Peer relationship and adolescent smartphone addiction: the mediating role of self-esteem and the moderating role of the need to belong. J Behav Addict.

[7] Marchant A, Hawton K, Stewart A, Montgomery P, Singaravelu V (2017). A systematic review of the relationship between internet use, self-harm and suicidal behaviour in young people: the good, the bad and the unknown. PLoS ONE.

[8] Grant JE, Lust K, Chamberlain SR (2019). Problematic smartphone use associated with greater alcohol consumption, mental health issues, poorer academic performance, and impulsivity. J Behav Addict.

[9] Müller M, Montag C (2017). The relationship between Internet addiction and alcohol consumption is influenced by the smoking status in male online video gamers. Clin Neuropsychiatry..

[10] Yang X, Zhou Z, Liu Q, Fan C (2019). Mobile phone addiction and adolescents’ anxiety and depression: the moderating role of mindfulness. J Child Fam Stud.

[11] Gao T, Li J, Zhang H, Gao J, Kong Y, Hu Y, Mei S (2018). The influence of alexithymia on mobile phone addiction: the role of depression, anxiety and stress. J Affect Disord.

[12] Przepiorka A, Blachnio A (2016). Time perspective in Internet and Facebook addiction. Comput Hum Behav.

[13] Davis RA (2001). A cognitive-behavioral model of pathological Internet use. Comput Hum Behav.

[14] Brand M, Wegmann E, Stark R, Müller A, Wölfling K, Robbins TW, Potenza MN (2019). The Interaction of Person-Affect-Cognition-Execution (I-PACE) model for addictive behaviors: update, generalization to addictive behaviors beyond Internet-use disorders, and specification of the process character of addictive behaviors. Neurosci Biobehav Rev.

[15] Chittaro L, Vianello A (2013). Time perspective as a predictor of problematic Internet use: a study of Facebook users. Personal Individ Differ.

[16] Gámez-Guadix M, Calvete E (2016). Assessing the relationship between mindful awareness and problematic Internet use among adolescents. Mindfulness..

[17] Billieux J (2012). Problematic use of the mobile phone: a literature review and a pathways model. Curr Psychiatry Rev.

[18] Acuff SF, Soltis KE, Dennhardt AA, Borsari B, Martens MP, Murphy JG (2017). Future so bright? Delay discounting and consideration of future consequences predict academic performance among college drinkers. Exp Clin Psychopharmacol.

[19] King DL, Delfabbro PH (2014). The cognitive psychology of Internet gaming disorder. Clin Psychol Rev.

[20] Komnenić D, Filipović S, Vukosavljević-Gvozden T (2015). Assessing maladaptive cognitions related to online gaming: proposing an adaptation of online cognitions scale. Comput Hum Behav.

[21] Wigfield A (1994). Expectancy-value theory of achievement motivation: a developmental perspective. Educ Psychol Rev.

[22] Park CJ, Hyun JS, Kim JY, Lee KE. Impact of personal time-related factors on smart phone addiction of female high school students. In: Proceedings of the world congress on engineering and computer science, vol. 1. 2014. p. 1–5.

[23] Liu LQ, Min G, Yue ST, Cheng LS (2018). The influence of mobile phone addiction on procrastination: a moderated mediating model. J Ergon.

[24] Vredenburg K, O’Brien E, Krames L (1988). Depression in college students: personality and experiential factors. J Counsel Psychol.

[25] Zimbardo PG, Boyd JN (1999). Putting time in perspective: a valid, reliable individual-differences metric. J Personal Soc Psychol.

[26] Lahey BB (2009). Public health significance of neuroticism. Am Psychol.

[27] Peterka-Bonetta J, Sindermann C, Elhai JD, Montag C (2019). Personality associations with smartphone and internet use disorder: a comparison study including links to impulsivity and social anxiety. Front Public Health.

[28] Gleeson JF, Rawlings D, Jackson HJ, Mcgorry PD (2005). Agreeableness and neuroticism as predictors of relapse after first-episode psychosis: a prospective follow-up study. J Nerv Ment Dis.

[29] Davies S, Filippopoulos P (2015). Changes in psychological time perspective during residential addiction treatment: a mixed-methods study. J Groups Addict Recov.

[30] Hoppmann CA, Infurna FJ, Ram N, Gerstorf D (2015). Associations among individuals’ perceptions of future time, individual resources, and subjective well-being in old age. J Gerontol B Psychol Sci Soc Sci.

[31] Baltes PB, Mayer KU. The Berlin aging study: aging from 70 to 100. Cambridge University Press, 2001.

[32] Diaconu-Gherasim LR, Bucci CM, Giuseppone KR, Brumariu LE (2017). Parenting and adolescents’ depressive symptoms: the mediating role of future time perspective. J Psychol.

[33] Boumosleh JM, Jaalouk D (2017). Depression, anxiety, and smartphone addiction in university students—a cross sectional study. PLoS ONE.

[34] Alhassan AA, Alqadhib EM, Taha NW, Alahmari RA, Salam M, Almutairi AF (2018). The relationship between addiction to smartphone usage and depression among adults: a cross sectional study. BMC Psychiatry..

[35] Montag C, Sindermann C, Becker B, Panksepp J (1906). An affective neuroscience framework for the molecular study of Internet addiction. Front Psychol.

[36] Elhai JD, Yang H, Montag C. Cognitive-and emotion-related dysfunctional coping processes: transdiagnostic mechanisms explaining depression and anxiety’s relations with problematic smartphone use. Current Addiction Reports 2019. p. 1–8.

[37] Bronfenbrenner U (1977). Lewinian space and ecological substance. J Soc Issues..

[38] Barnett E, Spruijt-Metz D, Unger JB, Rohrbach LA, Sun P, Sussman S (2013). Bidirectional associations between future time perspective and substance use among continuation high-school students. Subst Use Misuse..

[39] Kim J, Hong H, Lee J, Hyun MH (2017). Effects of time perspective and self-control on procrastination and Internet addiction. J Behav Addict.

[40] Zentsova NI, Leonov SV (2013). Comparative characteristics of time perspective of professional athletes and drug addicted people. Procedia Soc Behav Sci.

[41] Brown KW, Ryan RM (2003). The benefits of being present: mindfulness and its role in psychological well-being. J Personal Soc Psychol.

[42] Kabat-Zinn J (2003). Mindfulness-based interventions in context: past, present, and future. Clin Psychol Sci Pract.

[43] Baer RA, Smith GT, Hopkins J, Krietemeyer J, Toney L (2006). Using self-report assessment methods to explore facets of mindfulness. Assessment..

[44] Felder JN, Laraia B, Coleman-Phox K, Bush N, Suresh M, Thomas M, Prather AA (2018). Poor sleep quality, psychological distress, and the buffering effect of mindfulness training during pregnancy. Behav Sleep Med.

[45] Mathad MD, Rajesh SK, Pradhan B (2019). Spiritual well-being and its relationship with mindfulness, self-compassion and satisfaction with life in baccalaureate nursing students: a correlation study. J Relig Health.

[46] De Jong M, Peeters F, Gard T, Ashih H, Doorley J, Walker R, Hoge EA (2018). A randomized controlled pilot study on mindfulness-based cognitive therapy for unipolar depression in patients with chronic pain. J Clin Psychiatry..

[47] Elices M, Soler J, Feliu-Soler A, Carmona C, Tiana T, Pascual JC, Álvarez E (2017). Combining emotion regulation and mindfulness skills for preventing depression relapse: a randomized-controlled study. Borderline Personal Disord Emot Dysregul.

[48] Hunter L (2017). Mindfulness training can reduce depression and anxiety among nurses. Evid Based Nurs.

[49] Lindsay EK, Creswell JD (2017). Mechanisms of mindfulness training: monitor and Acceptance Theory (MAT). Clin Psychol Rev.

[50] Brown KW, Ryan RM, Creswell JD (2007). Mindfulness: theoretical foundations and evidence for its salutary effects. Psychol Inq.

[51] Andrews MC, Kacmar KM, Kacmar C (2014). The mediational effect of regulatory focus on the relationships between mindfulness and job satisfaction and turnover intentions. Career Dev Int.

[52] Higgins ET (1997). Beyond pleasure and pain. Am Psychol.

[53] Elhai JD, Levine JC, O’Brien KD, Armour C (2018). Distress tolerance and mindfulness mediate relations between depression and anxiety sensitivity with problematic smartphone use. Comput Hum Behav..

[54] Hundt NE, Kimbrel NA, Mitchell JT, Nelson-Gray RO (2008). High BAS, but not low BIS, predicts externalizing symptoms in adults. Personal Individ Differ.

[55] Khang H, Woo HJ, Kim JK (2011). Self as an antecedent of mobile phone addiction. Int J Mob Commun.

[56] Lyu H, Huang X (2016). Development and validation of future time perspective scale for adolescents and young adults. Time Soc.

[57] Dong-Ping LI. Future time perspective, goal orientation, social connectedness and undergraduates’ academic adjustment. Psychol Dev Educ. 2008 **(in Chinese)**.

[58] Gang D, Houchao L, Psychology FO, University S. The relationship of adolescents’ future time perspective and academic achievement: the mediation effect of time management disposition. J Psychol Sci. 2017 **(in Chinese)**.

[59] Leung L (2008). Linking psychological attributes to addiction and improper use of the mobile phone among adolescents in Hong Kong. J Child Media.

[60] Han L, Geng J, Jou M, Gao F, Yang H (2017). Relationship between shyness and mobile phone addiction in chinese young adults: mediating roles of self-control and attachment anxiety. Comput Hum Behav..

[61] Qing-Qi L, Dong-Jing Z, Xiu-Juan Y, Chen-Yan Z, Cui-Ying F, Zong-Kui Z (2018). Perceived stress and mobile phone addiction in chinese adolescents: a moderated mediation model. Comput Hum Behav.

[62] Lovibond PF, Lovibond SH (1995). The structure of negative emotional states: comparison of the Depression Anxiety Stress Scales (DASS) with the Beck Depression and Anxiety Inventories. Behav Res Ther.

[63] Gong X, Xie XY, Xu R, Luo YJ. Psychometric properties of the Chinese versions of DASS-21 in Chinese college students. Chin J Clin Psychol. 2010 **(in Chinese).**

[64] Greco LA, Baer RA, Smith GT (2011). Assessing mindfulness in children and adolescents: development and validation of the child and adolescent mindfulness measure (CAMM). Psychol Assess.

[65] de Bruin EI, Zijlstra BJ, Bögels SM (2014). The meaning of mindfulness in children and adolescents: further validation of the Child and Adolescent Mindfulness Measure (CAMM) in two independent samples from the Netherlands. Mindfulness..

[66] Liu QQ, Zhou ZK, Yang XJ, Kong FC, Niu GF, Fan CY (2017). Mobile phone addiction and sleep quality among chinese adolescents: a moderated mediation model. Comput Hum Behav.

[67] Zhou ZK, Liu QQ, Niu GF, Sun XJ, Fan CY (2017). Bullying victimization and depression in Chinese children: a moderated mediation model of resilience and mindfulness. Personal Individ Differ.

[68] MacKinnon DP (2008). Introduction to statistical mediation analysis.

[69] Hayes AF (2013). Introduction to mediation, moderation, and conditional process analysis: a regression-based approach.

[70] De Bilde J, Vansteenkiste M, Lens W (2011). Understanding the association between future time perspective and self-regulated learning through the lens of self-determination theory. Learn Instr.

[71] Miller RB, Brickman SJ (2004). A model of future-oriented motivation and self-regulation. Educ Psychol Rev.

[72] Carelli Maria Grazia, Olsson Carl-Johan (2014). Neural Correlates of Time Perspective. Time Perspective Theory; Review, Research and Application.

[73] Montag C, Becker M (2019). Psychological and neuroscientific advances to understand internet use disorder. Neuroforum..

[74] Montag Christian (2018). The Neuroscience of Smartphone/Social Media Usage and the Growing Need to Include Methods from ‘Psychoinformatics’. Information Systems and Neuroscience.

[75] Gindrat AD, Chytiris M, Balerna M, Rouiller EM, Ghosh A (2015). Use-dependent cortical processing from fingertips in touchscreen phone users. Curr Biol.

[76] Franck E, Vanderhasselt MA, Goubert L, Loeys T, Temmerman M, De Raedt R (2016). The role of self-esteem instability in the development of postnatal depression: a prospective study testing a diathesis-stress account. J Behav Ther Exp Psychiatry.

[77] Hankin BL, Abela JR, editors. Development of psychopathology: a vulnerability-stress perspective. New York: Sage Publications; 2005.

[78] Gao T, Xiang YT, Zhang H, Zhang Z, Mei S (2017). Neuroticism and quality of life: multiple mediating effects of smartphone addiction and depression. Psychiatry Res.

[79] Shumaker SA, Brownell A (1984). Toward a theory of social support: closing conceptual gaps. J Soc Issues.

[80] Skinner BF (1953). Science and human behavior.

[81] Chen J, Liang Y, Mai C, Zhong X, Qu C (2016). General deficit in inhibitory control of excessive smartphone users: evidence from an event-related potential study. Front Psychol.

[82] Bajaj B, Pande N (2016). Mediating role of resilience in the impact of mindfulness on life satisfaction and affect as indices of subjective well-being. Personal Individ Differ.

[83] Meiklejohn J, Phillips C, Freedman ML, Griffin ML, Biegel G, Roach A, Isberg R (2012). Integrating mindfulness training into K-12 education: fostering the resilience of teachers and students. Mindfulness..

[84] Agnew R, Brezina T, Wright JP, Cullen FT (2002). Strain, personality traits, and delinquency: extending general strain theory. Criminology..

[85] Wittmann M, Peter J, Gutina O, Otten S, Kohls N, Meissner K (2014). Individual differences in self-attributed mindfulness levels are related to the experience of time and cognitive self-control. Personal Individ Differ.

[86] Cluver L, Orkin M (2009). Cumulative risk and AIDS-orphanhood: interactions of stigma, bullying and poverty on child mental health in South Africa. Soc Sci Med.

[87] Sapacz M, Rockman G, Clark J (2016). Are we addicted to our cell phones?. Comput Hum Behav.

[88] Matousek RH, Dobkin PL, Pruessner J (2016). Cortisol as a marker for improvement in mindfulness-based stress reduction. Complement Ther Clin Pract.

[89] Davis TJ, Morris M, Drake MM (2016). The moderation effect of mindfulness on the relationship between adult attachment and wellbeing. Personal Individ Differ.

[90] Lin YH, Lin YC, Lee YH, Lin PH, Lin SH, Chang LR, Kuo TB (2015). Time distortion associated with smartphone addiction: identifying smartphone addiction via a mobile application (App). J Psychiatr Res.

[91] Montag C, Błaszkiewicz K, Lachmann B, Sariyska R, Andone I, Trendafilov B, Markowetz A (2015). Recorded behavior as a valuable resource for diagnostics in mobile phone addiction: evidence from psychoinformatics. Behav Sci.

[92] Montag C, Lachmann B, Herrlich M, Zweig K (2019). Addictive features of social media/messenger platforms and Freemium games against the background of psychological and economic theories. Int J Environ Res Public Health.

